# Endocannabinoid Signaling Contributes to Experience-Induced Increase of Synaptic Release Sites From Parvalbumin Interneurons in Mouse Visual Cortex

**DOI:** 10.3389/fncel.2020.571133

**Published:** 2020-09-30

**Authors:** Shiyong Huang, Alfredo Kirkwood

**Affiliations:** ^1^Program in Neuroscience, Hussman Institute for Autism, Baltimore, MD, United States; ^2^The Mind/Brain Institute, Johns Hopkins University, Baltimore, MD, United States; ^3^Department of Neuroscience, Johns Hopkins University, Baltimore, MD, United States

**Keywords:** parvalbumin interneuron, cannabinoid, postnatal development, connectivity, synaptic strength, perisomatic inhibition, visual cortex

## Abstract

During postnatal development of the visual cortex between eye-opening to puberty, visual experience promotes a gradual increase in the strength of inhibitory synaptic connections from parvalbumin-positive interneurons (PV-INs) onto layer 2/3 pyramidal cells. However, the detailed connectivity properties and molecular mechanisms underlying these developmental changes are not well understood. Using dual-patch clamp in brain slices from G42 mice, we revealed that both connection probability and the number of synaptic release sites contributed to the enhancement of synaptic strength. The increase of release site number was hindered by dark rearing from eye-opening and rescued by 3-days re-exposure to the normal visual environment. The effect of light re-exposure on restoring synaptic release sites in dark reared mice was mimicked by the agonist of cannabinoid-1 (CB1) receptors and blocked by an antagonist of these receptors, suggesting a role for endocannabinoid signaling in light-induced maturation of inhibitory connectivity from PV-INs to pyramidal cells during postnatal development.

## Introduction

Parvalbumin-expressing interneurons (PV-INs) are the prominent subtype of interneurons in the cortex (Rudy et al., 2011). Distinct anatomical and functional features allow PV-INs to provide fast feedforward and feedback inhibition. PV basket cells form perisomatic inhibitions onto pyramidal cells (Tremblay et al., 2016), highly connected with neighbor pyramidal cells and themselves (Holmgren et al., 2003; Packer and Yuste, 2011; Avermann et al., 2012; Gu et al., 2013; Pfeffer et al., 2013), and are uniquely poised to control network firing (Rudy et al., 2011; Bridi et al., 2020b).

PV-INs are crucial for several cortical functions. They are essential for gain control (Atallah et al., 2012), play a role on feature selectivity (Lee et al., 2012; Duan et al., 2017; Goel et al., 2018), participate in gamma rhythms (Cardin et al., 2009; Sohal et al., 2009). Importantly, in the visual cortex the protracted development of perisomatic inhibition has been implicated in the gating and control of ocular dominance plasticity during the critical period (Huang et al., 1999; Fagiolini et al., 2004; for reviews see Jiang et al., 2005; van Versendaal and Levelt, 2016; Choi, 2018; Hensch and Quinlan, 2018).

In the visual cortex, perisomatic inhibition develops postnatally and its maturation is completed at the beginning of puberty (~5 weeks of age). This maturation involves the increase in the number of synapses (Huang et al., 1999; Chattopadhyaya et al., 2004; Kreczko et al., 2009) and also an increase in the strength (Morales et al., 2002; Jiang et al., 2010a; Tatti et al., 2017). There is abundant evidence that this process depends on visual experience (Benevento et al., 1995; Morales et al., 2002; Chattopadhyaya et al., 2004; Katagiri et al., 2007; Kreczko et al., 2009; Jiang et al., 2010b). However, the underlying molecular mechanisms and its consequences for the functional circuitry are not clear. Here we show that the experience developmental increase in the strength of perisomatic inhibition reflects the increase in the number of PV-IN→Pyr connections and in the number of synapses per connection, without changes in the quantal size of quantal content. This change can be mimicked by the agonist of CB1 receptors and blocked by an antagonist of CB1 receptors. Our findings provide evidence that endocannabinoid may be involved in experience induced maturation of inhibitory synapse number during development.

## Materials and Methods

### Animals

G42 mice were obtained directly from Dr. J. Z. Huang (Cold Spring Harbor Laboratory) *via* Dr. Bernardo Rudy. Offspring were produced from homozygous or heterozygous breeding pairs. Either sex of mice was used in this study. Normally reared mice were reared in a 12-h light/dark cycles. Dark reared mice were reared in a dark room with light completely blocked. I.P. injections in dark reared mice were performed with the help of a night vision goggle in the darkroom. All procedures were approved by the Institutional Animal Care and Use Committee at the Johns Hopkins University.

### Brain Slice Preparation

Acute visual cortical slices (300 μm) from G42 mice were prepared as previously described (Jiang et al., 2010b; Gu et al., 2013; Huang et al., 2013; Gao et al., 2017; Bridi et al., 2020b). Briefly, slices were cut in the ice-cold dissection buffer containing (in mM): 212.7 sucrose, 5 KCl, 1.25 NaH_2_PO_4_, 10 MgCl_2_, 0.5 CaCl_2_, 26 NaHCO_3_, 10 dextrose, bubbled with 95% O_2_/5% CO_2_ (pH 7.4). Slices were then transferred into a recovery chamber containing normal artificial cerebrospinal fluid (ACSF) and recovered for 30 min in a 30°C water bath. After that, the slices were recovered in the room temperature for at least an additional 30 min before recording. Normal ACSF was similar to the dissection buffer except that the concentrations of MgCl_2_ and CaCl_2_ were 1 mM and 2 mM, respectively, and sucrose was replaced by NaCl (124 mM).

### Dual-Patch Clamp

**Figure 1:**
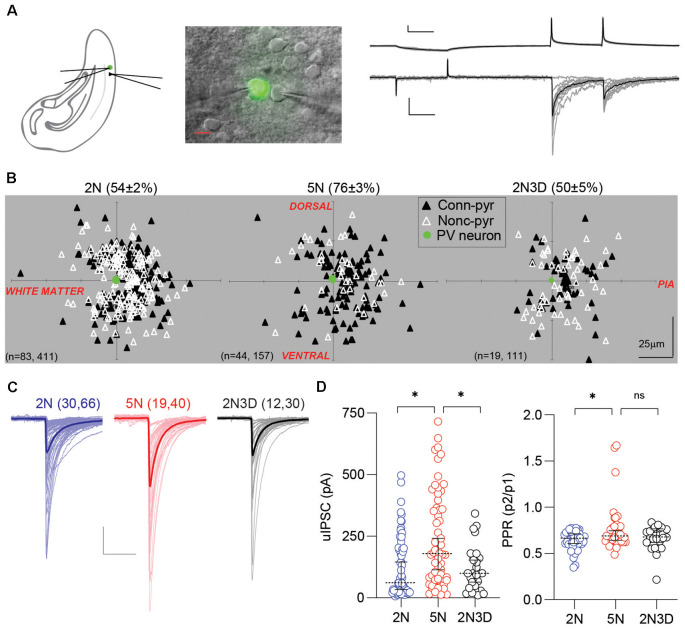
Experience-dependent maturation of PV-IN→Pyr synaptic connectivity. **(A)** Configuration of dual patch-clamp recording in the layer 2/3 visual cortex of G42 mice expressing GFP in PV-INs. Example of 10 consecutive unitary inhibitory postsynaptic currents (uIPSCs) recorded in the pyramidal cell in response to action potentials elicited in the presynaptic PV-IN. Individual responses in gray, average in black. Scale bars represent 20 mV, 100 pA, and 50 ms. **(B)** Connection probability and relative positions of PV-INs (green filled circles) and pyramidal cells (triangles) in layer 2/3 visual cortical slices from normal-reared 2-week-old mice (2N), normal-reared 5-week-old mice (5N), or 5-week-old mice reared in the dark when 2 weeks old (2N3D). Black triangles represent connected PV-IN→Pyr pairs, white triangles represent non-connected pairs. **(C)** Each superimposed trace represents the averaged uIPSCs (15 sweeps) from a connected pair recorded in 2N, 5N, and 2N3D mice. Thick traces represent the group’s grand average. Scale bars represent 100 pA and 50 ms. **(D)** Summary graphs depicting the uIPSC amplitude, on the left, and the pair-pulse ratio (PPR) recorded in the three rearing-experience groups. Circles represent individual cases, horizontal bars indicate the median with 95% CI. The number of mice and cell pairs is indicated in parentheses in **(B,C)**. Asterisks indicate *p* < 0.05. ns: not significant.

Two types of internal solution were used for dual-patch clamp in this study: a K-based internal solution (in mM: 130 KGluconate, 10 KCl, 0.2 EGTA, 10 HEPES, 0.5 NaGTP, 4 MgATP, and 10 Na_2_-Phosphocreatine; pH adjusted to 7.25 with KOH, 280–290 mOsm), and a CsCl-based internal solution (in mM: 120 CsCl, 8 NaCl, 10 HEPES, 2 EGTA, 5 QX-314, 0.5 Na_2_GTP, 4 MgATP, and 10 Na_2_-Phosphocreatine; pH adjusted to 7.25 with CsOH, 280–290 mOsm). As previously described (Jiang et al., 2010b; Gu et al., 2013; Huang et al., 2013; Gao et al., 2017; Bridi et al., 2020b), in the layer 2/3 of the visual cortex, PV-INs were identified by fluorescence and recorded under current clamp with pipettes containing the K-based internal solution. Pyramidal cells were visually identified by their pyramidal looking soma and clear apical dendrite (Jiang et al., 2007) and recorded under voltage clamp with pipettes filled with the CsCl-based internal solution. Patch-clamp recordings were performed at 30°C. Only cells with membrane potentials <−60 mV, series resistance <20 MΩ were studied. Patch-clamp properties were monitored with 100-ms negative voltage or current commands (−2 mV and −40 pA for voltage and current clamp, respectively) delivered every 20–30 s. Cells were excluded if series resistance changed >15% over the experiment. Data were filtered at 2 kHz and digitized at 10 kHz using Igor Pro (WaveMetrics). Synaptic currents were recorded from pyramidal cells holding at −60 mV in the presence of 20 μM 6-cyano-7-nitroquinoxaline-2,3-dione (CNQX) and 100 μM 2-amino-5-phosphonovaleric acid (APV) and evoked PV-INs firing every 20 s by a depolarized current with 2 ms pulses delivered in pairs [inter-stimulus interval (ISI): 100 ms] to compute paired-pulse ratio (PPR = P2/P1, where P1 and P2 are the amplitudes of the responses to the first and second stimulation, respectively; Figure 1A). Spike features in PV-INs were calculated as previously described (Bridi et al., 2017). The distance between the cell pair of PV-IN and pyramidal cell and the position of the cell pairs in the brain slices were recorded.

### Chemicals and I.P. Injection

WIN 55,212-2 (WIN), AM251, DL-AP5, and CNQX were purchased from Sigma and the R&D system. CB1 receptor antagonist and agonist, WIN and AM251 were dissolved in the vehicle solution [10% Tween 80, 20% DMSO, and 70% saline (0.9% NaCl)] to a final concentration of 1 mg/ml for systematic injection (I.P.). To minimize variability in these experiments the control and treated mice came from the same litter and were housed together. On the day of the experiment we tested one control and one treated mice and the order of sacrifice was alternated every day.

### Data Analysis and Statistics

The mean-variance analysis was performed on responses evoked by 15 stimulus trains (5 or 10 stimuli at 30–50 Hz) delivered at 20 s intervals. The unitary inhibitory postsynaptic current (uIPSC) amplitude was measured for each stimulus, and the mean (*I*) and variance (*var*) were plotted against each other. Synaptic parameters including the number of release sites (*N*) and the quantal size (*q*) were obtained by fitting the data to the parabola: *var = q.I−I^2^/N* as previously described (Scheuss and Neher, 2001; Gu et al., 2013). We previously validated using this approach to evaluate parameters in PV-IN→Pyr connections (Gu et al., 2013). We considered only those cases in which the R^2^ value of the fit was >0.5.

For comparison in connection probability, a *χ*^2^ test was applied to determine variation, and a Fishers Exact *p*-value was used to determine the significance. For other two-group comparisons, significance was examined by unpaired two-tailed *t*-tests or Mann–Whitney (M–W) tests based on the normality of dataset using the D’Agostino-Pearson omnibus normality test. For multiple groups (more than two) comparisons, one way ANOVA test followed by Holm–Sidak’s multiple comparisons test (normally distributed data), or Kruskal–Wallis test followed by Dunn’s multiple comparisons test (non-normal distributed data) were used to determine statistical significance. *P* < 0.05 was considered as statistical significance. Statistical outliers in the amplitude of uIPSCs were detected using pre-established criteria (ROUT test) and excluded from analysis (Supplementary Table S1).

## Results

During postnatal development, in the period between eye-opening to puberty, visual experience promotes a ~3-fold increase in the strength of GABAergic perisomatic inhibition onto layer 2/3 pyramidal cell (Huang et al., 1999; Morales et al., 2002; Chattopadhyaya et al., 2004; Lu et al., 2014). It is unclear whether this increase in perisomatic inhibition reflects an increase in connectivity, i.e., more interneurons contacting a given pyramidal cell, an increase in the potency of these contacts, or both. Since parvalbumin-positive inhibitory interneurons (PV-INs) contribute the majority of perisomatic inhibitory inputs (Kruglikov and Rudy, 2008), we addressed these questions by performing paired recordings of pyramidal cells (Pyr) and PV-INs in the layer 2/3 of acute visual cortical slices of G42 mice, a line that expresses GFP in PV-INs (Chattopadhyaya et al., 2004).

### Experience-Dependent Developmental Increase in PV-IN→Pyr Connectivity

We compared the PV-IN→Pyr connectivity in visual cortical slices from three groups of G42 mice that differed in age and visual experience: normally-reared 2-week-old mice (2N, postnatal days 14–16), normally-reared 5-week-old mice (5N, postnatal days 35–37) and 5-week-old mice reared in the dark when 2 weeks old (2N3D, postnatal days 35–37). Action potentials were induced in the PV-INs and the uIPSCs were recorded in pyramidal cells (see “Materials and Methods” section; Figure 1A). As shown in Figure 1B, the connection probability of the PV-IN→Pyr pairs increased significantly between 2 and 5 weeks of age in normal reared mice (2N: 0.54 ± 0.02, *n* = 411 pairs, 83 mice; 5N: 0.76 ± 0.04, *n* = 157 pairs, 44 mice; *p* < 0.0001; Fisher’s exact test), and this developmental increase did not occur in mice reared in the dark for 3 weeks (2N3D: 0.50 ± 0.05, *n* = 111 pairs, 19 mice; 5N vs. 2N3D: *p* < 0.0001; 2N vs. 2N3D: *p* = 0.5205; Fisher’s exact test). Similarly, in connected PV-IN→Pyr pairs the amplitude of uIPSCs also increased from postnatal 2 weeks to 5 weeks in normally reared mice, and it did not occur in mice reared in the dark (statistic value = 15.53, *p* = 0.0004, Kruskal–Wallis test; 2N vs. 5N: *p* = 0.0004; 5N vs. 2N3D: *p* = 0.0176; Dunn’s multiple comparison test; Figures 1C,D). We also measured the PPR (P2/P1) of the uIPSCs as an indicator of presynaptic release probability. The PPR of uIPSCs was larger in 5N mice compared to 2N mice but visual deprivation after eye-opening did not significantly affect this increase (statistic value = 5.55, *p* = 0.0623, Kruskal–Wallis test; 2N vs. 5N: *p* = 0.0383; 5N vs. 2N3D: *p* = 0.8013; Dunn’s multiple comparison; Figure 1D). These results indicate that during postnatal maturation, PV-INs connect with more nearby pyramidal cells and with stronger connections. Those changes require visual experience.

### Developmental Increase of uIPSCs Amplitude Reflects Release Sites per PV-IN→Pyr Connection

**Figure 2:**
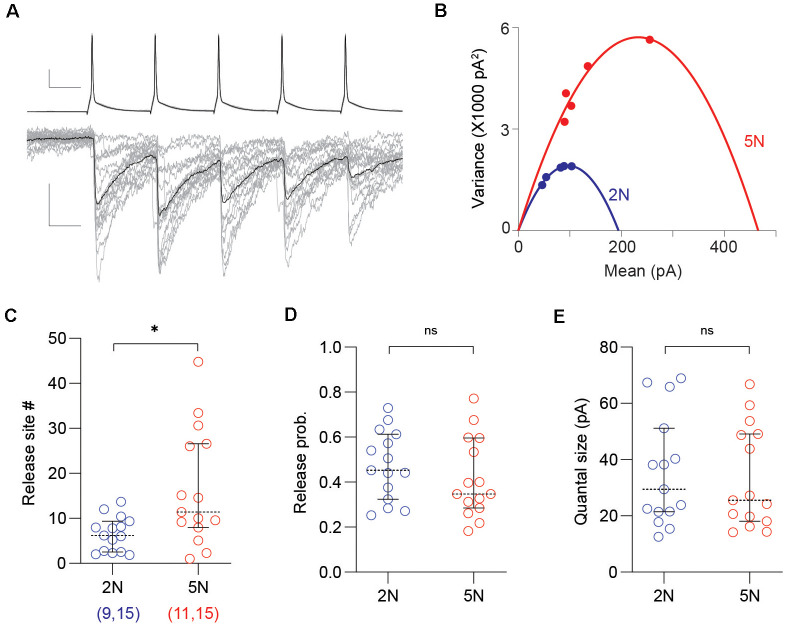
Mean-variance analysis reveals a developmental increase of release sites per PV-IN→Pyr connection. **(A)** Representative uIPSC evoked by 50 Hz trains (of five pulses) used in the mean-variance analysis to estimate synaptic parameters. Superimposed traces are 15 consecutive sweeps (gray) and the averaged response (black). Scale bars represent 20 mV, 100 pA, and 10 ms. **(B)** The relationship between mean amplitude and variance of the five uIPSCs in the trains from 2N (blue) and 5N (red) mice were fitted with parabolas. **(C–E)** Summary graphs depicting synaptic parameters including the number of release sites **(C)**, the release probability **(D)**, and the quantal size **(E)** estimated from the parabolic fit (see “Materials and Methods” section) in 2N and 5N mice. Circles represent individual cases, horizontal bars indicate the median with 95% CI. The number of mice and analyzed cell pairs are indicated in parentheses. Asterisks indicate *p* < 0.05. ns: not significant.

To further understand the maturation of synaptic properties in connected PV-IN→Pyr cell pairs, we used mean-variance analysis to estimate the quantal parameters from uIPSCs, including the number of the synaptic release site, the release probability, and the quantal size (Figures 2A,B). This approach was validated in our previous publication (Gu et al., 2013). We found that the number of release site significantly increased from 2N to 5N (2N: *n* = 15 pairs, nine mice; 5N: *n* = 15 pairs, 11 mice; *t*_(28)_ = 2.889, *p* = 0.0074, unpaired *t*-test; Figure 2C), but not the release probability (*t*_(28)_ = 0.9500, *p* = 0.3502, unpaired *t*-test; Figure 2D) or the quantal size (*t*_(28)_ = 0.3182, *p* = 0.7527, unpaired *t*-test; Figure 2E). These results indicated that the developmental increase in the uIPSC amplitude can be attributed to an increase in the number of release sites, probably reflecting more synaptic contacts per PV-IN→Pyr pairs.

### Light Exposure *via* Endocannabinoid Signaling Increases the uIPSC Amplitude and the Number of Synaptic Release Sites in Dark-Reared Mice

**Figure 3:**
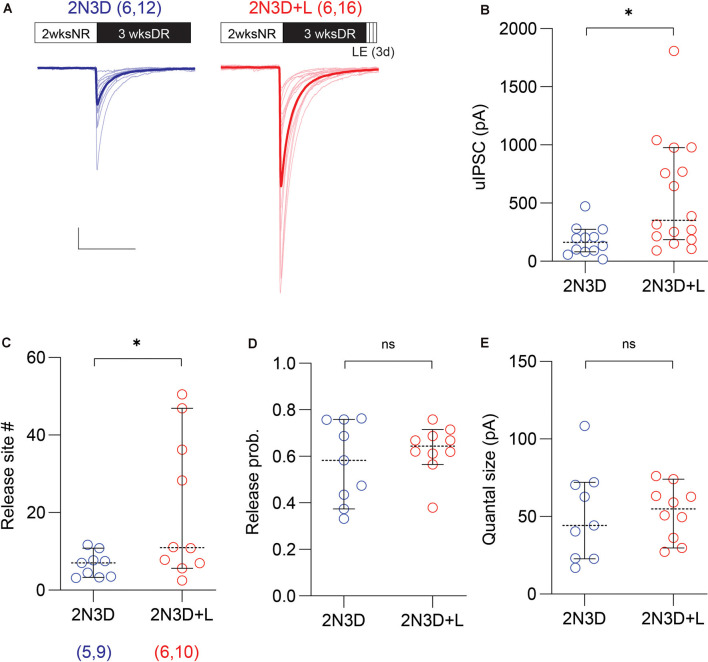
Light exposure after dark rearing increases the uIPSC amplitude and the number of synaptic release sites. **(A)** Superimposed traces are uIPSCs from 2N3D mice (blue) and 2N3D mice exposed to light for 3 days (2N3D + L: red) and their group average (thick lines). Scale bars: 100 pA and 50 ms. **(B)** Larger uIPSCs amplitude in 2N3D mice exposed to the normal light environment for 3 days. **(C–E)** The number of release sites **(C)** but not the release probability **(D)** or the quantal size **(E)** increased after the light exposure. Circles represent individual cases, horizontal bars indicate the median with 95% CI. The number of mice and analyzed cell pairs are indicated in parentheses. Asterisks indicate *p* < 0.05. ns: not significant.

Previously we showed that brief exposure to normal light environment can rescue alterations in GABAergic release induced by prolonged dark-rearing (Jiang et al., 2010b). We asked, therefore, whether a brief exposure to light can restore the strength of PV-IN→Pyr connectivity in 2N3D mice. In six light-exposed mice (2N3D + L), the proportion of connected pairs was still low (only 17 out of 34 connected pairs) and comparable to the proportion observed in six non-exposed 2N3D mice (12 out of 32 pairs; *p* = 0.3325, Fisher exact test). In contrast, the exposure to light did increase significantly the amplitude of uIPSCs in the connected PV-IN→Pyr pairs (2N3D + L: 559.7 ± 118.1 pA, *n* = 16 pairs, six mice; 2N3D: 176.3 ± 36.3 pA, *n* = 12 pairs, six mice; *U* = 38, *p* = 0.0061, M-W test; Figures 3A,B). In a subset of these connected PV-IN→Pyr cell pairs we performed the Mean-variance analysis as showed in Figure 2. We found that light exposure increased the number of release sites (2N3D + L: 20.7 ± 5.7, *n* = 10 pairs; 2N3D: 6.6 ± 1.1, *n* = 9 pairs; *t*_(17)_ = 2.288, *p* = 0.0352, unpaired *t*-test; Figure 3C), but did not change the release probability (2N3D + L: 0.63 ± 0.03; 2N3D: 0.57 ± 0.06; *U* = 40, *p* = 0.7197, M–W test; Figure 3D) or quantal size (2N3D + L: 52.8 ± 5.5 pA; 2N3D: 51.3 ± 10.0 pA; *t*_(17)_ = 0.1470, *p* = 0.8849, unpaired *t*-test; Figure 3E).

**Figure 4:**
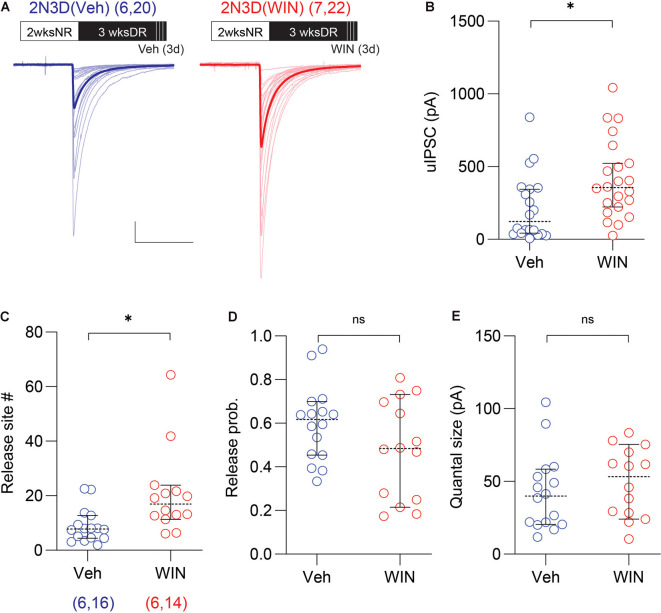
Cannabinoid agonist mimics light exposure in increasing the uIPSC amplitude and the number of synaptic release sites. **(A)** Superimposed traces are uIPSCs from 2N3D mice systemically injected with the cannabinoid-1 (CB1) receptor agonist WIN 55,212-2 (WIN; 10 mg/kg, I.P.: red) or vehicle (Veh: blue) for 3 days. Group grand averages are depicted with thick lines. Scale bars: 100 pA and 50 ms. **(B)** Larger uIPSCs amplitude in 2N3D mice injected with WIN. **(C–E)** The number of release sites **(C)**, but not the release probability **(D)**, or the quantal size **(E)** increased after the systematic administration of WIN. Circles represent individual cases, horizontal bars indicate the median with 95% CI. The number of mice and analyzed cell pairs are indicated in parentheses. Asterisks indicate *p* < 0.05. ns: not significant.

Next, we explored possible molecular mechanisms contributing to the rapid recovery of the uIPSC amplitude and the number of release sites induced by light exposure. Previously we reported that endocannabinoid signaling plays a role in the experience-dependent maturation of short-term plasticity of GABAergic synaptic transmission in the layer 2/3 visual cortex (Jiang et al., 2010a,b; Sun et al., 2015). Therefore, we examined the role of endocannabinoid signaling in the rapid recovery of the uIPSCs amplitude and the number of release sites induced by light re-exposure in the dark reared mice. To that end, we tested whether the effects of light exposure can be respectively mimicked and prevented by cannabinoid agonists and antagonists. First, we evaluated in the 2N3D mice the effects of the agonist of cannabinoid-1 (CB1) receptor WIN injected systemically in the last 3 days of dark rearing (10 mg/kg, I.P. injection twice per day). We found that in 2N3D mice injected with WIN, the uIPSC amplitude was larger than that in the control 2N3D mice injected with vehicle (2N3D + WIN: 411.3 ± 57.1 pA, *n* = 22 pairs, seven mice; 2N3D + Veh: 218.0 ± 50.4 pA, *n* = 20 pairs, six mice; *U* = 120, *p* = 0.0111, M–W test; Figures 4A,B. Similarly, the variance analysis revealed a larger number of synaptic release sites in the WIN-injected 2N3D mice (2N3D + WIN: 20.6 ± 4.1, *n* = 14 pairs; 2N3D + Veh: 8.9 ± 1.6, *n* = 16 pairs; *U* = 44, *p* = 0.0039, M–W test; Figure 4C), but no differences either in the quantal size (2N3D + WIN: 49.3 ± 6.4 pA; 2N3D + Veh: 42.5 ± 6.6 pA; *t*_(28)_ = 0.7307, *p* = 0.4710, unpaired *t*-test; Figure 4E) or in the computed release probability (2N3D + WIN: 0.48 ± 0.06; 2N3D + Veh: 0.60 ± 0.04; *t*_(28)_ = 1.658, *p* = 0.1085, unpaired *t*-test; Figure 4D). We also found that WIN injection did not change the spike features or membrane properties in PV-INs or pyramidal cells (Supplementary Table S2).

**Figure 5:**
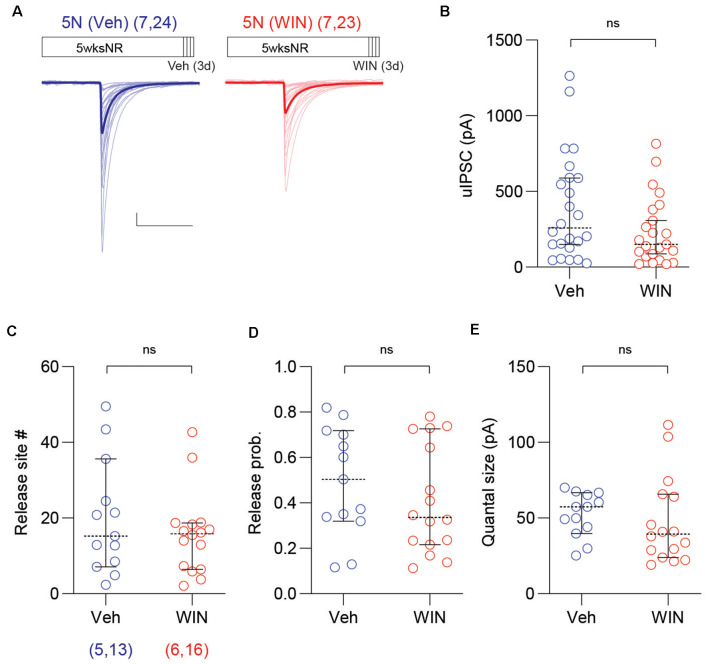
Cannabinoid agonist does not affect uIPSC of mature normal reared mice. **(A)** Superimposed traces are uIPSCs from 5N mice systemically injected with the CB1 receptor agonist WIN (10 mg/kg, I.P.: red) or vehicle (Veh: blue) for 3 days. Group grand averages are depicted with thick lines. Scale bars: 100 pA and 50 ms. **(B)** The amplitude of uIPSCs did not change in 5N mice injected with WIN for 3 days. **(C–E)** The number of release sites **(C)**, the release probability **(D)**, or the quantal size **(E)** did not differ after the WIN injection in 5N mice. Circles represent individual cases, horizontal bars indicate the median with 95% CI. The number of mice and analyzed cell pairs are indicated in parentheses. Asterisks indicate *p* < 0.05. ns: not significant.

We considered the possibility that WIN injections may alter the PV-IN→Pyr connectivity in normally reared mice. To that end, we repeated the 3-day WIN treatment (10 mg/kg, I.P. injection twice per day) in 5N mice, and found that in WIN injected mice the proportion of connected PV-INs→Pyr pairs was comparable to that of 5N mice injected with vehicle (5N + WIN: 26 out of 35 pairs; 5N + Veh: 26 out of 35 pairs; *p* > 0.9999, Fisher exact test). In a similar manner, there were no differences in the uIPSC amplitude (5N + WIN: 238.3 ± 46.6 pA, *n* = 23 pairs, seven mice; 5N + Veh: 390.1 ± 71.2 pA, *n* = 24 pairs, seven mice; *U* = 200, *p* = 0.1086, M–W test; Figures 5A,B), the number of release site (5N + WIN: 15.8 ± 2.7, *n* = 16 pairs; 5N + Veh: 19.9 ± 4.1, *n* = 13 pairs; *U* = 91, *p* = 0.5890, M–W test; Figure 5C), the release probability (5N + WIN: 0.41 ± 0.06; 5N + Veh: 0.49 ± 0.07; *t*_(27)_ = 0.9182, *p* = 0.3666, unpaired *t*-test; Figure 5D), or the quantal size (5N + WIN: 47.7 ± 7.2 pA; 5N + Veh: 52.6 ± 4.0 pA; *t*_(27)_ = 0.5484, *p* = 0.5879, unpaired *t*-test; Figure 5E), after 3-day WIN injection in 5-weeks-old normally reared mice. The WIN injection in 5N mice modestly affected intrinsic firing properties: it decreased the spike threshold voltage, trough voltage, and upstroke/downstroke ratio, and increased the input resistance and spike downstroke of PV-INs (Supplementary Table S3).

**Figure 6:**
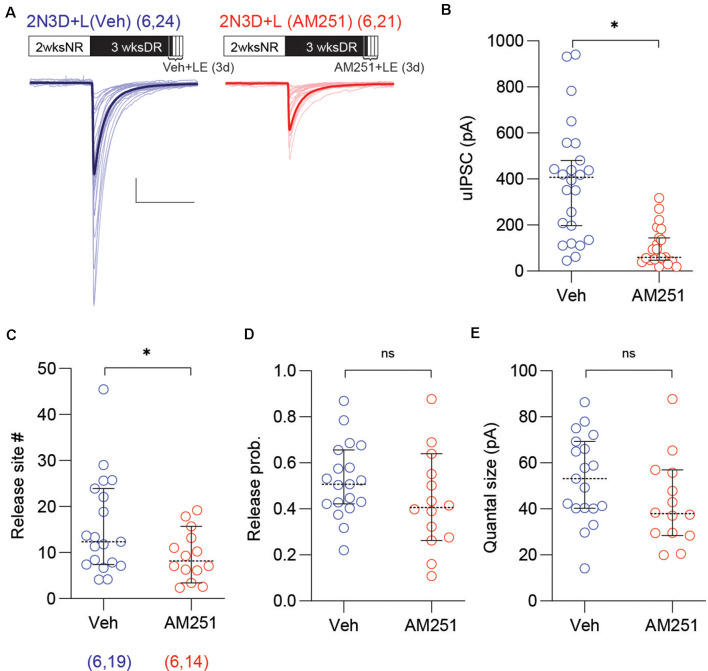
The antagonist of the CB1 receptor prevents the increase of the uIPSC amplitude and the number of synaptic release sites by light exposure. **(A)** Superimposed traces are uIPSCs from 2N3D + L mice injected either with the CB1 receptor antagonist AM251 (10 mg/kg, I.P.: red) or vehicle (blue) for 3 days. Group grand averages are depicted with thick lines. Scale bars: 100 pA and 50 ms. **(B)** The amplitude of uIPSCs is smaller in 2N3D + L mice treated with AM251. **(C–E)** The number of release sites **(C)** but not the release probability **(D)** or the quantal size **(E)** is smaller in the AM251 treated group. Circles represent individual cases, horizontal bars indicate the median with 95% CI. The number of mice and analyzed cell pairs are indicated in parentheses. Asterisks indicate *p* < 0.05. ns: not significant.

Finally, in a complementary set of studies, we tested whether the CB1 antagonist AM251 prevents the increase of PV-IN→Pyr strength in 2N3D mice re-exposed to normal light/dark cycles for 3 days. In these 2N3D + L mice, we injected AM251 daily during the light exposure (10 mg/kg, I.P. injection twice per day, started 1 day before the light re-exposure). Compared to vehicle-injected mice, the mice injected with AM251 showed smaller uIPSC amplitude (2N3D + L + AM251: 106.4 ± 18.7 pA, *n* = 21 pairs, six mice; 2N3D + L + Veh: 391.6 ± 52.2 pA, *n* = 24 pairs, six mice; *U* = 64, *p* < 0.0001, M–W test; Figures 6A,B), and a smaller number of release sites (2N3D + L + AM251: 9.4 ± 1.5 pA, *n* = 14 pairs; 2N3D + L + Veh: 15.8 ± 2.4 pA, *n* = 19 pairs; *U* = 78, *p* = 0.0461, M–W test; Figure 6C), but without changes in either the release probability (2N3D + L + AM251: 0.43 ± 0.06; 2N3D + L + Veh: 0.52 ± 0.04; *t*_(31)_ = 1.43, *p* = 0.1626, unpaired *t*-test; Figure 6D) or the quantal size (2N3D + L + AM251: 42.7 ± 5.0 pA; 2N3D + L + Veh: 53.3 ± 4.3 pA; *t*_(31)_ = 1.599, *p* = 0.1199, unpaired *t*-test; Figure 6E). Altogether the results indicate that after a long period of dark rearing, the reduced strength of the PV-IN→Pyr connections, but not the lowered probability of connections, can be rapidly restored by brief exposure to light, in a process that can be mimicked by CB1 agonists, and prevented by CB1 antagonists. The AM251 injections did not change the spike features or membrane properties in PV-INs or pyramidal cells (Supplementary Table S4).

## Discussion

Our analysis of PV-IN circuitry in the G42 line revealed that the potency and abundance of PV-IN→Pyr connections both increase during postnatal development. Dark rearing since eye-opening arrest both processes, and in the deprived mice the potency of PV-IN→Pyr inputs, although not the connectivity levels, can be rapidly restored by brief light exposure. We surmise that at the quantal level, the developmental/experience increase in potency reflects the addition of release sites without overt changes in release probability or quantal size, in a process that requires cannabinoid signaling.

PV-INs were identified based on the fluorescence in acute brain slices of G42 mice in this study. These mice are BAC transgenic mice that express GFP under GAD67 gene promoters exclusively in PV-INs (Chattopadhyaya et al., 2004), but only in a subset of them (Chattopadhyaya et al., 2004; Sippy and Yuste, 2013; Large et al., 2016). PV-positive interneurons are comprised of two subtypes of fast-spiking interneurons, basket cells, and chandelier cells, with distinct anatomical and functional features (Povysheva et al., 2013; Taniguchi et al., 2013; Inan and Anderson, 2014; Miyamae et al., 2017). Although chandelier cells are far less common than basket cells, we can’t rule out that we recorded a mixture of these two subtypes of fast-spiking interneurons.

We found that the connection probability and the unitary strength of PV-IN→Pyr connections in layer 2/3 cortex increased during development, which is in agreement with previous reports of a protracted maturation of PV-IN circuits in the visual cortex (Chattopadhyaya et al., 2004; Lazarus and Huang, 2011; Lu et al., 2014), dentate gyrus (Doischer et al., 2008), and prefrontal cortex (Yang et al., 2014). The increase in input strength resulted from the increased number of synaptic release sites rather than quantal size or release probability. The mean-variance analysis indicated a 2-fold increase in synaptic release sites per PV-IN→Pyr connection from the 2nd to the 5th week (from ~10 to ~20 release sites; Figure 2B). On the other hand, the PV-IN→Pyr connection probability increases 1.5 times from 2-week-old to 5-week-old (from ~50% to ~75%; Figure 1B), which should yield a ~3-fold increase in the total number release sites onto each pyramidal cell during this developmental period. This estimation matches the 3-fold developmental increase in the magnitude of the maximal evoked IPSCs in pyramidal cells (Morales et al., 2002), and it also matches the ~3-fold developmental increase in perisomatic boutons onto pyramidal cells reported in anatomical studies (Chattopadhyaya et al., 2004; Kreczko et al., 2009). Notably, the developmental increase in all of these measures of GABAergic strength, number of release sites, maximal IPSC, and number perisomatic GABAergic boutons, can be arrested early dark rearing. There are apparent discrepancies, however. In adult mice, each pyramidal cell in layer 2/3 visual cortex has connections from about 14 PV-INs (Safari et al., 2017). Considering an average of ~20 release sites per connection (Figure 2), this would yield ~280 release sites per adult pyramidal cells, which far exceed ~22 perisomatic GAD puncta observes in the adult layer 2/3 pyramidal cells (Kreczko et al., 2009). This may indicate multivesicular release in PV-IN inhibitory synapses, as reported in hippocampal GABAergic synapses, where there is a fivefold discrepancy between the numbers of functionally determined release sites and structurally identified active zones (Biro et al., 2006). Finally, it is worth pointing out that from a functional point of view, increases in release sites without changes in release probability could be a way to strengthen inhibitory output without altering the dynamic properties of release, which in the case of PV-INs, are uniquely tuned to maintain the performance over a wide range of firing activity (Bridi et al., 2020b). In turn, this idea concords with the notion of a need for mechanisms that change overall network inhibition without compromising the temporal dynamics of synaptic inhibition (Gao et al., 2017).

Our results with CB1 ligands support a role for endocannabinoid signaling in the visually-driven increase of PV-IN→Pyr release sites. This is consistent with studies showing altered PN-Ins circuits in CB1 KO mice (Fitzgerald et al., 2012; Aso et al., 2018). The underlying mechanisms remain unknown. Early immunohistochemistry studies reported that CB1 and parvalbumin are expressed in the largely distinct non-overlapping interneuron population (Katona et al., 1999; Wedzony and Chocyk, 2009), which argues against a direct cannabinoid action on PV-INs. Later studies, however, did report CB1 expression in PV-INs in a variety of brain structures (McDonald and Mascagni, 2001; Fusco et al., 2004; Freiman et al., 2006; Narushima et al., 2006; Uchigashima et al., 2007; Horne et al., 2013; Rivera et al., 2014; Holley et al., 2019). Moreover, CB1 agonists do affect PV-IN synaptic transmission in the cortex (Jiang et al., 2010b). Nevertheless, indirect mechanisms are also plausible. For example, activation of CB1 receptors on glia can affect synaptic transmission between neurons (reviewed by Castillo et al., 2012; Metna-Laurent and Marsicano, 2015). Similarly, systemic delivery of cannabinoids increases the BDNF levels in PFC and hippocampus (Sales et al., 2019), and BDNF is a key mediator in CB1 receptor-dependent protection against excitotoxicity (Khaspekov et al., 2004).

Multiple mechanisms have been implicated in the maturation of inhibitory synapses made PV-INs. Some of them promote maturation, like BDNF (Huang et al., 1999; Gianfranceschi et al., 2003; Jiao et al., 2011). GABA (Chattopadhyaya et al., 2007), OTX2 (Sugiyama et al., 2008), NPAS4 (Lin et al., 2008), SynGAP1 (Berryer et al., 2016) while others, such as HDAC1 (Koh and Sng, 2016), polysialic acid (PSA; Di Cristo et al., 2007) and p75 neurotrophin receptor (p75NR; Baho et al., 2019) negatively regulate the maturation of inhibitory synapses from PV-INs. Our study provides evidence for the role of endocannabinoid signaling in the experience-dependent increase of release sites in PV-INs.

Although acute applications of CB1 agonists promote long-term depression of GABAergic transmission (reviewed by Chevaleyre et al., 2006), the current observation that systemic and prolonged exposure to cannabinoids strengthen PV-IN→Pyr output complements previous findings that cannabinoids increase the fidelity of PV-IN→Pyr transmission (Jiang et al., 2010b) and play a role in the sleep-dependent enhancement of GABAergic inhibition (Bridi et al., 2020a). The positive effect of cannabinoids on the number of release sites makes this mechanism an attractive target to increase the strength of GABAergic circuitry for therapeutic purposes. Unlike other signaling mechanisms that promote PV-IN output, like BDNF for example, cortical CB1 receptors can be readily accessed by small molecules capable of crossing the blood-brain barrier. In this context, it will be of interest to determine whether the beneficial effects of cannabidiol in models of Alzheimer’s disease (Aso et al., 2018) and autism spectrum disorder (Fleury-Teixeira et al., 2019) are partly due to an increase in PV-IN release sites.

## Data Availability Statement

The datasets for generating figures of this study are available in the Supplementary Materials.

## Ethics Statement

The animal study was reviewed and approved by IACUC at the Johns Hopkins University.

## Author Contributions

SH conducted experiments and analyzed data. SH and AK designed experiments, interpreted results, and wrote the manuscript.

## Conflict of Interest

The authors declare that the research was conducted in the absence of any commercial or financial relationships that could be construed as a potential conflict of interest.

## References

[B1] Aso E. Andrés-Benito P. Ferrer I. (2018). Genetic deletion of CB_1_ cannabinoid receptors exacerbates the Alzheimer-like symptoms in a transgenic animal model. Biochem. Pharmacol. 157, 210–216. 10.1016/j.bcp.2018.08.007 30096288

[B2] Atallah B. V. Bruns W. Carandini M. Scanziani M. (2012). Parvalbumin-expressing interneurons linearly transform cortical responses to visual stimuli. Neuron 73, 159–170. 10.1016/j.neuron.2011.12.013 22243754 PMC3743079

[B3] Avermann M. Tomm C. Mateo C. Gerstner W. Petersen C. C. H. (2012). Microcircuits of excitatory and inhibitory neurons in layer 2/3 of mouse barrel cortex. J. Neurophysiol. 107, 3116–3134. 10.1152/jn.00917.2011 22402650

[B4] Baho E. Chattopadhyaya B. Lavertu-Jolin M. Mazziotti R. Awad P. N. Chehrazi P.. (2019). p75 neurotrophin receptor activation regulates the timing of the maturation of cortical parvalbumin interneuron connectivity and promotes juvenile-like plasticity in adult visual cortex. J. Neurosci. 39, 4489–4510. 10.1523/jneurosci.2881-18.2019 30936240 PMC6554620

[B5] Benevento L. A. Bakkum B. W. Cohen R. S. (1995). Gamma-aminobutyric acid and somatostatin immunoreactivity in the visual cortex of normal and dark-reared rats. Brain Res. 689, 172–182. 10.1016/0006-8993(95)00553-3 7583320

[B6] Berryer M. H. Chattopadhyaya B. Xing P. Riebe I. Bosoi C. Sanon N.. (2016). Decrease of SYNGAP1 in GABAergic cells impairs inhibitory synapse connectivity, synaptic inhibition and cognitive function. Nat. Commun. 7:13340. 10.1038/ncomms13340 27827368 PMC5105197

[B7] Biro A. A. Holderith N. B. Nusser Z. (2006). Release probability-dependent scaling of the postsynaptic responses at single hippocampal GABAergic synapses. J. Neurosci. 26, 12487–12496. 10.1523/jneurosci.3106-06.2006 17135411 PMC2630420

[B8] Bridi M. C. D. Zong F.-J. Min X. Luo N. Tran T. Qiu J.. (2020a). Daily oscillation of the excitation-inhibition balance in visual cortical circuits. Neuron 105, 621.e4–629.e4. 10.1016/j.neuron.2019.11.011 31831331 PMC9520672

[B9] Bridi M. S. Park S. M. Huang S. (2017). Developmental disruption of GABA_A_R-meditated inhibition in Cntnap2 KO mice. eNeuro 4:ENEURO.0162-17.2017. 10.1523/eneuro.0162-17.2017 28966979 PMC5617210

[B10] Bridi M. S. Shin S. Huang S. Kirkwood A. (2020b). Dynamic recovery from depression enables rate encoding in inhibitory synapses. iScience 23:100940. 10.1016/j.isci.2020.100940 32163896 PMC7066227

[B11] Cardin J. A. Carlén M. Meletis K. Knoblich U. Zhang F. Deisseroth K.. (2009). Driving fast-spiking cells induces gamma rhythm and controls sensory responses. Nature 459, 663–667. 10.1038/nature08002 19396156 PMC3655711

[B12] Castillo P. E. Younts T. J. Chavez A. E. Hashimotodani Y. (2012). Endocannabinoid signaling and synaptic function. Neuron 76, 70–81. 10.1016/j.neuron.2012.09.020 23040807 PMC3517813

[B13] Chattopadhyaya B. Di Cristo G. Higashiyama H. Knott G. W. Kuhlman S. J. Welker E.. (2004). Experience and activity-dependent maturation of perisomatic GABAergic innervation in primary visual cortex during a postnatal critical period. J. Neurosci. 24, 9598–9611. 10.1523/jneurosci.1851-04.2004 15509747 PMC6730138

[B14] Chattopadhyaya B. Di Cristo G. Wu C. Z. Knott G. Kuhlman S. Fu Y.. (2007). GAD67-mediated GABA synthesis and signaling regulate inhibitory synaptic innervation in the visual cortex. Neuron 54, 889–903. 10.1016/j.neuron.2007.05.015 17582330 PMC2077924

[B15] Chevaleyre V. Takahashi K. A. Castillo P. E. (2006). Endocannabinoid-mediated synaptic plasticity in the CNS. Annu. Rev. Neurosci. 29, 37–76. 10.1146/annurev.neuro.29.051605.112834 16776579

[B16] Choi S.-Y. (2018). Synaptic and circuit development of the primary sensory cortex. Exp. Mol. Med. 50:13. 10.1038/s12276-018-0029-x 29628505 PMC5938038

[B17] Di Cristo G. Chattopadhyaya B. Kuhlman S. J. Fu Y. Bélanger M.-C. Wu C. Z.. (2007). Activity-dependent PSA expression regulates inhibitory maturation and onset of critical period plasticity. Nat. Neurosci. 10, 1569–1577. 10.1038/nn2008 18026099

[B18] Doischer D. Hosp J. A. Yanagawa Y. Obata K. Jonas P. Vida I.. (2008). Postnatal differentiation of basket cells from slow to fast signaling devices. J. Neurosci. 28, 12956–12968. 10.1523/jneurosci.2890-08.2008 19036989 PMC6671784

[B19] Duan J. Fu H. Zhang J. (2017). Activation of parvalbumin-positive neurons in both retina and primary visual cortex improves the feature-selectivity of primary visual cortex neurons. Neurosci. Bull. 33, 255–263. 10.1007/s12264-016-0096-8 28074441 PMC5567512

[B20] Fagiolini M. Fritschy J.-M. Löw K. Möhler H. Rudolph U. Hensch T. K. (2004). Specific GABAA circuits for visual cortical plasticity. Science 303, 1681–1683. 10.1126/science.1091032 15017002

[B21] Fitzgerald M. L. Chan J. Mackie K. Lupica C. R. Pickel V. M. (2012). Altered dendritic distribution of dopamine D2 receptors and reduction in mitochondrial number in parvalbumin-containing interneurons in the medial prefrontal cortex of cannabinoid-1 (CB1) receptor knockout mice. J. Comp. Neurol. 520, 4013–4031. 10.1002/cne.23141 22592925 PMC3685630

[B22] Fleury-Teixeira P. Caixeta F. V. Ramires da Silva L. C. Brasil-Neto J. P. Malcher-Lopes R. (2019). Effects of CBD-enriched cannabis sativa extract on autism spectrum disorder symptoms: an observational study of 18 participants undergoing compassionate use. Front. Neurol. 10:1145. 10.3389/fneur.2019.01145 31736860 PMC6834767

[B23] Freiman I. Anton A. Monyer H. Urbanski M. J. Szabo B. (2006). Analysis of the effects of cannabinoids on identified synaptic connections in the caudate-putamen by paired recordings in transgenic mice. J. Physiol. 575, 789–806. 10.1113/jphysiol.2006.114272 16825300 PMC1995699

[B24] Fusco F. R. Martorana A. Giampá C. De March Z. Farini D. D’Angelo V.. (2004). Immunolocalization of CB1 receptor in rat striatal neurons: a confocal microscopy study. Synapse 53, 159–167. 10.1002/syn.20047 15236348

[B25] Gao M. Whitt J. L. Huang S. Lee A. Mihalas S. Kirkwood A.. (2017). Experience-dependent homeostasis of “noise” at inhibitory synapses preserves information coding in adult visual cortex. Philos. Trans. R. Soc. Lond. B Biol. Sci. 372:20160156. 10.1098/rstb.2016.0156 28093550 PMC5247588

[B26] Gianfranceschi L. Siciliano R. Walls J. Morales B. Kirkwood A. Huang Z. J.. (2003). Visual cortex is rescued from the effects of dark rearing by overexpression of BDNF. Proc. Natl. Acad. Sci. U S A 100, 12486–12491. 10.1073/pnas.1934836100 14514885 PMC218784

[B27] Goel A. Cantu D. A. Guilfoyle J. Chaudhari G. R. Newadkar A. Todisco B.. (2018). Impaired perceptual learning in a mouse model of fragile X syndrome is mediated by parvalbumin neuron dysfunction and is reversible. Nat. Neurosci. 21, 1404–1411. 10.1038/s41593-018-0231-0 30250263 PMC6161491

[B28] Gu Y. Huang S. Chang M. C. Worley P. Kirkwood A. Quinlan E. M. (2013). Obligatory role for the immediate early gene NARP in critical period plasticity. Neuron 79, 335–346. 10.1016/j.neuron.2013.05.016 23889936 PMC3804028

[B29] Hensch T. K. Quinlan E. M. (2018). Critical periods in amblyopia. Vis. Neurosci. 35:E014. 10.1017/S0952523817000219 29905116 PMC6047524

[B30] Holley S. M. Galvan L. Kamdjou T. Dong A. Levine M. S. Cepeda C. (2019). Major contribution of somatostatin-expressing interneurons and cannabinoid receptors to increased GABA synaptic activity in the striatum of huntington’s disease mice. Front. Synaptic Neurosci. 11:14. 10.3389/fnsyn.2019.00014 31139071 PMC6527892

[B31] Holmgren C. Harkany T. Svennenfors B. Zilberter Y. (2003). Pyramidal cell communication within local networks in layer 2/3 of rat neocortex. J. Physiol. 551, 139–153. 10.1113/jphysiol.2003.044784 12813147 PMC2343144

[B32] Horne E. A. Coy J. Swinney K. Fung S. Cherry A. E. T. Marrs W. R.. (2013). Downregulation of cannabinoid receptor 1 from neuropeptide Y interneurons in the basal ganglia of patients with Huntington’s disease and mouse models. Eur. J. Neurosci. 37, 429–440. 10.1111/ejn.12045 23167744 PMC3699342

[B33] Huang S. Huganir R. L. Kirkwood A. (2013). Adrenergic gating of Hebbian spike-timing-dependent plasticity in cortical interneurons. J. Neurosci. 33, 13171–13178. 10.1523/jneurosci.5741-12.2013 23926270 PMC3735889

[B34] Huang Z. J. Kirkwood A. Pizzorusso T. Porciatti V. Morales B. Bear M. F.. (1999). BDNF regulates the maturation of inhibition and the critical period of plasticity in mouse visual cortex. Cell 98, 739–755. 10.1016/s0092-8674(00)81509-3 10499792

[B35] Inan M. Anderson S. A. (2014). The chandelier cell, form and function. Curr. Opin. Neurobiol. 26, 142–148. 10.1016/j.conb.2014.01.009 24556285 PMC4024324

[B36] Jiang B. Huang S. de Pasquale R. Millman D. Song L. Lee H. K.. (2010b). The maturation of GABAergic transmission in visual cortex requires endocannabinoid-mediated LTD of inhibitory inputs during a critical period. Neuron 66, 248–259. 10.1016/j.neuron.2010.03.021 20435001 PMC2897012

[B37] Jiang B. Huang Z. J. Morales B. Kirkwood A. (2005). Maturation of GABAergic transmission and the timing of plasticity in visual cortex. Brain Res. Rev. 50, 126–133. 10.1016/j.brainresrev.2005.05.007 16024085

[B38] Jiang B. Sohya K. Sarihi A. Yanagawa Y. Tsumoto T. (2010a). Laminar-specific maturation of GABAergic transmission and susceptibility to visual deprivation are related to endocannabinoid sensitivity in mouse visual cortex. J. Neurosci. 30, 14261–14272. 10.1523/JNEUROSCI.2979-10.2010 20962247 PMC6634750

[B39] Jiang B. Trevino M. Kirkwood A. (2007). Sequential development of long-term potentiation and depression in different layers of the mouse visual cortex. J. Neurosci. 27, 9648–9652. 10.1523/jneurosci.2655-07.2007 17804625 PMC6672979

[B40] Jiao Y. Zhang Z. Zhang C. Wang X. Sakata K. Lu B.. (2011). A key mechanism underlying sensory experience-dependent maturation of neocortical GABAergic circuits *in vivo*. Proc. Natl. Acad. Sci. U S A 108, 12131–12136. 10.1073/pnas.1105296108 21730187 PMC3141955

[B41] Katagiri H. Fagiolini M. Hensch T. K. (2007). Optimization of somatic inhibition at critical period onset in mouse visual cortex. Neuron 53, 805–812. 10.1016/j.neuron.2007.02.026 17359916

[B42] Katona I. Sperlágh B. Sík A. Käfalvi A. Vizi E. S. Mackie K.. (1999). Presynaptically located CB1 cannabinoid receptors regulate GABA release from axon terminals of specific hippocampal interneurons. J. Neurosci. 19, 4544–4558. 10.1523/jneurosci.19-11-04544.1999 10341254 PMC6782612

[B43] Khaspekov L. G. Brenz Verca M. S. Frumkina L. E. Hermann H. Marsicano G. Lutz B. (2004). Involvement of brain-derived neurotrophic factor in cannabinoid receptor-dependent protection against excitotoxicity. Eur. J. Neurosci. 19, 1691–1698. 10.1111/j.1460-9568.2004.03285.x 15078543

[B44] Koh D. X. P. Sng J. C. G. (2016). HDAC1 negatively regulates Bdnf and Pvalb required for parvalbumin interneuron maturation in an experience-dependent manner. J. Neurochem. 139, 369–380. 10.1111/jnc.13773 27534825

[B45] Kreczko A. Goel A. Song L. Lee H.-K. (2009). Visual deprivation decreases somatic GAD65 puncta number on layer 2/3 pyramidal neurons in mouse visual cortex. Neural. Plast. 2009:415135. 10.1155/2009/415135 19503840 PMC2686200

[B46] Kruglikov I. Rudy B. (2008). Perisomatic GABA release and thalamocortical integration onto neocortical excitatory cells are regulated by neuromodulators. Neuron 58, 911–924. 10.1016/j.neuron.2008.04.024 18579081 PMC2572574

[B47] Large A. M. Kunz N. A. Mielo S. L. Oswald A.-M. M. (2016). Inhibition by somatostatin interneurons in olfactory cortex. Front. Neural Circuits 10:62. 10.3389/fncir.2016.00062 27582691 PMC4987344

[B48] Lazarus M. S. Huang Z. J. (2011). Distinct maturation profiles of perisomatic and dendritic targeting GABAergic interneurons in the mouse primary visual cortex during the critical period of ocular dominance plasticity. J. Neurophysiol. 106, 775–787. 10.1152/jn.00729.2010 21613595 PMC3154827

[B49] Lee S.-H. Kwan A. C. Zhang S. Phoumthipphavong V. Flannery J. G. Masmanidis S. C.. (2012). Activation of specific interneurons improves V1 feature selectivity and visual perception. Nature 488, 379–383. 10.1038/nature11312 22878719 PMC3422431

[B50] Lin Y. Bloodgood B. L. Hauser J. L. Lapan A. D. Koon A. C. Kim T. K.. (2008). Activity-dependent regulation of inhibitory synapse development by Npas4. Nature 455, 1198–1204. 10.1038/nature07319 18815592 PMC2637532

[B51] Lu J. Tucciarone J. Lin Y. Huang Z. J. (2014). Input-specific maturation of synaptic dynamics of parvalbumin interneurons in primary visual cortex. Proc. Natl. Acad. Sci. U S A 111, 16895–16900. 10.1073/pnas.1400694111 25385583 PMC4250102

[B52] McDonald A. J. Mascagni F. (2001). Localization of the CB1 type cannabinoid receptor in the rat basolateral amygdala: high concentrations in a subpopulation of cholecystokinin-containing interneurons. Neuroscience 107, 641–652. 10.1016/s0306-4522(01)00380-3 11720787

[B53] Metna-Laurent M. Marsicano G. (2015). Rising stars: modulation of brain functions by astroglial type-1 cannabinoid receptors. Glia 63, 353–364. 10.1002/glia.22773 25452006

[B54] Miyamae T. Chen K. Lewis D. A. Gonzalez-Burgos G. (2017). Distinct physiological maturation of parvalbumin-positive neuron subtypes in mouse prefrontal cortex. J. Neurosci. 37, 4883–4902. 10.1523/jneurosci.3325-16.2017 28408413 PMC5426180

[B55] Morales B. Choi S.-Y. Kirkwood A. (2002). Dark rearing alters the development of GABAergic transmission in visual cortex. J. Neurosci. 22, 8084–8090. 10.1523/jneurosci.22-18-08084.2002 12223562 PMC6758086

[B56] Narushima M. Uchigashima M. Hashimoto K. Watanabe M. Kano M. (2006). Depolarization-induced suppression of inhibition mediated by endocannabinoids at synapses from fast-spiking interneurons to medium spiny neurons in the striatum. Eur. J. Neurosci. 24, 2246–2252. 10.1111/j.1460-9568.2006.05119.x 17042791

[B57] Packer A. M. Yuste R. (2011). Dense, unspecific connectivity of neocortical parvalbumin-positive interneurons: a canonical microcircuit for inhibition. J. Neurosci. 31, 13260–13271. 10.1523/jneurosci.3131-11.2011 21917809 PMC3178964

[B58] Pfeffer C. K. Xue M. He M. Huang Z. J. Scanziani M. (2013). Inhibition of inhibition in visual cortex: the logic of connections between molecularly distinct interneurons. Nat. Neurosci. 16, 1068–1076. 10.1038/nn.3446 23817549 PMC3729586

[B59] Povysheva N. V. Zaitsev A. V. Gonzalez-Burgos G. Lewis D. A. (2013). Electrophysiological heterogeneity of fast-spiking interneurons: chandelier versus basket cells. PLoS One 8:e70553. 10.1371/journal.pone.0070553 23950961 PMC3741302

[B60] Rivera P. Arrabal S. Cifuentes M. Grondona J. M. Pérez-Martín M. Rubio L.. (2014). Localization of the cannabinoid CB1 receptor and the 2-AG synthesizing (DAGLα) and degrading (MAGL, FAAH) enzymes in cells expressing the Ca^(2+)^-binding proteins calbindin, calretinin and parvalbumin in the adult rat hippocampus. Front. Neuroanat. 8:56. 10.3389/fnana.2014.00056 25018703 PMC4073216

[B61] Rudy B. Fishell G. Lee S. Hjerling-Leffler J. (2011). Three groups of interneurons account for nearly 100% of neocortical GABAergic neurons. Dev. Neurobiol. 71, 45–61. 10.1002/dneu.20853 21154909 PMC3556905

[B62] Safari M. S. Mirnajafi-Zadeh J. Hioki H. Tsumoto T. (2017). Parvalbumin-expressing interneurons can act solo while somatostatin-expressing interneurons act in chorus in most cases on cortical pyramidal cells. Sci. Rep. 7:12764. 10.1038/s41598-017-12958-4 28986578 PMC5630625

[B63] Sales A. J. Fogaça M. V. Sartim A. G. Pereira V. S. Wegener G. Guimaraes F. S.. (2019). Cannabidiol induces rapid and sustained antidepressant-like effects through increased BDNF signaling and synaptogenesis in the prefrontal cortex. Mol. Neurobiol. 56, 1070–1081. 10.1007/s12035-018-1143-4 29869197

[B64] Scheuss V. Neher E. (2001). Estimating synaptic parameters from mean, variance and covariance in trains of synaptic responses. Biophys. J. 81, 1970–1989. 10.1016/s0006-3495(01)75848-1 11566771 PMC1301672

[B65] Sippy T. Yuste R. (2013). Decorrelating action of inhibition in neocortical networks. J. Neurosci. 33, 9813–9830. 10.1523/jneurosci.4579-12.2013 23739978 PMC3715137

[B66] Sohal V. S. Zhang F. Yizhar O. Deisseroth K. (2009). Parvalbumin neurons and gamma rhythms enhance cortical circuit performance. Nature 459, 698–702. 10.1038/nature07991 19396159 PMC3969859

[B67] Sugiyama S. Di Nardo A. A. Aizawa S. Matsuo I. Volovitch M. Prochiantz A.. (2008). Experience-dependent transfer of Otx2 homeoprotein into the visual cortex activates postnatal plasticity. Cell 134, 508–520. 10.1016/j.cell.2008.05.054 18692473

[B68] Sun W. Wang L. Li S. Tie X. Jiang B. (2015). Layer-specific endocannabinoid-mediated long-term depression of GABAergic neurotransmission onto principal neurons in mouse visual cortex. Eur. J. Neurosci. 42, 1952–1965. 10.1111/ejn.12958 25997857

[B69] Taniguchi H. Lu J. Huang Z. J. (2013). The spatial and temporal origin of chandelier cells in mouse neocortex. Science 339, 70–74. 10.1126/science.1227622 23180771 PMC4017638

[B70] Tatti R. Swanson O. K. Lee M. S. E. Maffei A. (2017). Layer-specific developmental changes in excitation and inhibition in rat primary visual cortex. eNeuro 4:ENEURO.0402-17.2017. 10.1523/eneuro.0402-17.2017 29379869 PMC5779119

[B71] Tremblay R. Lee S. Rudy B. (2016). GABAergic interneurons in the neocortex: from cellular properties to circuits. Neuron 91, 260–292. 10.1016/j.neuron.2016.06.033 27477017 PMC4980915

[B72] Uchigashima M. Narushima M. Fukaya M. Katona I. Kano M. Watanabe M. (2007). Subcellular arrangement of molecules for 2-arachidonoyl-glycerol-mediated retrograde signaling and its physiological contribution to synaptic modulation in the striatum. J. Neurosci. 27, 3663–3676. 10.1523/jneurosci.0448-07.2007 17409230 PMC6672418

[B73] van Versendaal D. Levelt C. N. (2016). Inhibitory interneurons in visual cortical plasticity. Cell. Mol. Life Sci. 73, 3677–3691. 10.1007/s00018-016-2264-4 27193323 PMC5002041

[B74] Wedzony K. Chocyk A. (2009). Cannabinoid CB1 receptors in rat medial prefrontal cortex are colocalized with calbindin- but not parvalbumin- and calretinin-positive GABA-ergic neurons. Pharmacol. Rep. 61, 1000–1007. 10.1016/s1734-1140(09)70161-6 20081234

[B75] Yang J. M. Zhang J. Yu Y. Q. Duan S. Li X. M. (2014). Postnatal development of 2 microcircuits involving fast-spiking interneurons in the mouse prefrontal cortex. Cereb. Cortex 24, 98–109. 10.1093/cercor/bhs291 23042741

