# Supplementary material for: Endocannabinoid Signaling Contributes to Experience-Induced Increase of Synaptic Release Sites From Parvalbumin Interneurons in Mouse Visual Cortex
Source: Front Cell Neurosci. 2020 Sep 30;14:571133. doi: 10.3389/fncel.2020.571133 (PMC7556304; doi:10.3389/fncel.2020.571133)
Supplement: Supplementary file 1 [file Table_1.pdf]

Table 1. Outliers detected and excluded in the amplitude of uIPSCs.

Figure 1C			Figure 3	Figure 4	Figure 5		Figure 6	
**2N**	**5N**	**2N3D**			**5N+Veh**	**5N+WIN**	**2N3DL+Veh**	**2N3DL+AM251**
511.3	1093.7	593.2	None	None	None	1153.4	None	706.6
513.4		536.1				1633.2		507.7
								586.2
								497.4
								736.7

Table 2. Membrane properties and spike features of PV-INs and Pyr-cells in 2N3D mice.

	Veh	WIN	Stat-value	
**V_spike peak (mV)**	17.3 ± 2.2	16.2 ± 1.8	$t_{(20)}$ = 0.3674	p = 0.7172
**V_trough (mV)**	-62.7 ± 1.9	-66.8 ± 1.0	U = 39	p = 0.2093
**V_threshold (mV)**	-51.0 ± 2.0	-53.1 ± 0.9	$t_{(20)}$ = 0.8392	p = 0.4113
**Spike height (mV)**	80.1 ± 1.8	83.0 ± 2.1	$t_{(20)}$ = 1.053	p = 0.3049
**Spike width (ms)**	0.44 ± 0.03	0.45 ± 0.03	U = 57.5	p = 0.9576
**Upstroke (mV/ms)**	347.7 ± 25.7	370.9 ± 30.6	$t_{(20)}$ = 0.5801	p = 0.5683
**Downstroke (mV/ms)**	-226.4 ± 17.5	-220.5 ± 22.0	$t_{(20)}$ = 0.2132	p = 0.8333
**Up/downstroke ratio**	-1.59 ± 0.11	-1.72 ± 0.07	$t_{(20)}$ = 0.9076	p = 0.3749
**RMP (mV)**	-68.4 ± 1.0	-69.6 ± 1.1	$t_{(20)}$ = 0.7618	p = 0.4551
**Rin (MΩ)**	81.5 ± 8.3	70.5 ± 10.3	$t_{(14)}$ = 0.8165	p = 0.4279
**Rin_Pyr (MΩ)**	360.7 ± 45.4	341.4 ± 26.6	U = 221	p = 0.8193

Two-tailed unpaired t test or M-W test reported with *t* or *U*, respectively, was used for statistical analyses. Data were presented as mean ± SEM.

Table 3. Membrane properties and spike features of PV-INs and Pyr-cells in 5N mice.

	Veh	WIN	Stat-value	
**V_spike peak (mV)**	13.9 ± 1.9	13.3 ± 2.1	$t_{(23)} = 0.1993$	$p = 0.8438$
**V_trough (mV)**	-67.1 ± 0.5	-63.5 ± 0.6	$t_{(23)} = 4.395$	$\mathbf{p = 0.0002}$
**V_threshold (mV)**	-52.7 ± 1.0	-46.3 ± 2.3	$t_{(23)} = 2.272$	$\mathbf{p = 0.0328}$
**Spike height (mV)**	80.0 ± 2.0	76.8 ± 2.1	$t_{(23)} = 1.461$	$p = 0.1575$
**Spike width (ms)**	0.38 ± 0.03	0.50 ± 0.06	$U = 43$	$p = 0.0566$
**Upstroke (mV/ms)**	378.6 ± 27.6	324.2 ± 24.1	$t_{(23)} = 1.485$	$p = 0.1511$
**Downstroke (mV/ms)**	-248.8 ± 17.7	-189.9 ± 18.9	$t_{(23)} = 2.224$	$\mathbf{p = 0.0362}$
**Up/downstroke ratio**	-1.52 ± 0.04	-1.77 ± 0.08	$U = 31$	$\mathbf{p = 0.0108}$
**RMP (mV)**	-70.0 ± 0.8	-67.8 ± 0.8	$U = 48.5$	$p = 0.1177$
**Rin (MΩ)**	53.1 ± 3.3	66.1 ± 4.2	$t_{(23)} = 2.353$	$\mathbf{p = 0.0275}$
**Rin_Pyr (MΩ)**	272.5 ± 24.8	313.2 ± 33.6	$t_{(48)} = 0.9724$	$p = 0.3357$

Two-tailed unpaired t test or M-W test reported with *t* or *U*, respectively, was used for statistical analyses. Data were presented as mean ± SEM. Bold *p* value indicates statistical significance. Data were presented as mean ± SEM.

Table 4. Membrane properties and spike features of PV-INs and Pyr-cells in 2N3D+L mice.

	**Veh**	**AM251**	**Stat-value**	
**V_spike peak (mV)**	17.0 ± 1.9	15.1 ± 2.1	$U = 80$	$p = 0.4274$
**V_trough (mV)**	-65.7 ± 1.0	-64.5 ± 0.8	$U = 74$	$p = 0.2852$
**V_threshold (mV)**	-48.8 ± 1.0	-47.9 ± 2.0	$U = 96$	$p = 0.9459$
**Spike height (mV)**	81.8 ± 1.8	79.7 ± 2.0	$U = 92$	$p = 0.5907$
**Spike width (ms)**	0.44 ± 0.02	0.55 ± 0.09	$U = 104$	$p = 0.9732$
**Upstroke (mV/ms)**	366.9 ± 17.2	366.0 ± 26.3	$t_{(27)} = 0.0302$	$p = 0.9761$
**Downstroke (mV/ms)**	-211.9 ± 13.7	-205.6 ± 20.2	$t_{(23)} = 0.2629$	$p = 0.7946$
**Up/downstroke ratio**	-1.78 ± 0.08	-1.92 ± 0.14	$U = 97$	$p = 0.7472$
**RMP (mV)**	-69.1 ± 0.6	-68.3 ± 0.8	$t_{(26)} = 0.7766$	$p = 0.4444$
**Rin (MΩ)**	68.3 ± 6.2	77.7 ± 6.8	$U = 73$	$p = 0.1718$
**Rin_Pyr (MΩ)**	352.3 ± 38.4	355.7 ± 52.7	$U = 271$	$p = 0.4350$

Two-tailed unpaired t test or M-W test reported with *t* or *U*, respectively, was used for statistical analyses. Data were presented as mean ± SEM.
